# Molecular Network Analysis Reveals Transmission of HIV-1 Drug-Resistant Strains Among Newly Diagnosed HIV-1 Infections in a Moderately HIV Endemic City in China

**DOI:** 10.3389/fmicb.2021.797771

**Published:** 2022-01-07

**Authors:** Bin Zhao, Wei Song, Mingming Kang, Xue Dong, Xin Li, Lu Wang, Jianmin Liu, Haibo Ding, Zhenxing Chu, Lin Wang, Yu Qiu, Hong Shang, Xiaoxu Han

**Affiliations:** ^1^NHC Key Laboratory of AIDS Immunology, National Clinical Research Center for Laboratory Medicine, The First Affiliated Hospital of China Medical University, China Medical University, Shenyang, China; ^2^Laboratory Medicine Innovation Unit, Chinese Academy of Medical Sciences, Shenyang, China; ^3^Key Laboratory of AIDS Immunology of Liaoning Province, Shenyang, China; ^4^Collaborative Innovation Center for Diagnosis and Treatment of Infectious Diseases, Hangzhou, China; ^5^Department of Food Safety and Nutrition, Shenyang Center for Health Service and Administrative Law Enforcement, Shenyang Center for Disease Control and Prevention, Shenyang, China

**Keywords:** HIV, transmitted drug resistance (TDR), subtype, molecular network, China

## Abstract

Since the implementation of the “treat all” policy in China in 2016, there have been few data on the prevalence of transmitted drug resistance (TDR) in China. In this study, we describe TDR in patients newly diagnosed with human immunodeficiency virus (HIV) infection between 2016 and 2019 in Shenyang city, China. Demographic information and plasma samples from all newly reported HIV-infected individuals in Shenyang from 2016 to 2019 were collected. The HIV *pol* gene was amplified and sequenced for subtyping and TDR. The spread of TDR was analyzed by inferring an HIV molecular network based on pairwise genetic distance. In total, 2,882 sequences including CRF01_AE (2019/2,882, 70.0%), CRF07_BC (526/2,882, 18.3%), subtype B (132/2,882, 4.6%), and other subtypes (205/2,882, 7.1%) were obtained. The overall prevalence of TDR was 9.1% [95% confidence interval (CI): 8.1–10.2%]; the prevalence of TDR in each subtype in descending order was CRF07_BC [14.6% (95% CI: 11.7–18.0%)], subtype B [9.1% (95% CI: 4.8–15.3%)], CRF01_AE [7.9% (95% CI: 6.7–9.1%)], and other sequences [7.3% (95% CI: 4.2–11.8%)]. TDR mutations detected in more than 10 cases were Q58E (*n* = 51), M46ILV (*n* = 46), K103N (*n* = 26), E138AGKQ (*n* = 25), K103R/V179D (*n* = 20), and A98G (*n* = 12). Molecular network analysis revealed three CRF07_BC clusters with TDR [two with Q58E (29/29) and one with K103N (10/19)]; and five CRF01_AE clusters with TDR [two with M46L (6/6), one with A98G (4/4), one with E138A (3/3), and one with K103R/V179D (3/3)]. In the TDR clusters, 96.4% (53/55) of individuals were men who have sex with men (MSM). These results indicate that TDR is moderately prevalent in Shenyang (5–15%) and that TDR strains are mainly transmitted among MSM, providing precise targets for interventions in China.

## Introduction

In 2016, China modified the human immunodeficiency virus (HIV) antiretroviral therapy (ART) enrollment criteria according to World Health Organization (WHO) guidelines (World Health Organization, 2016) to include all HIV-infected patients regardless of CD4 cell counts (General Office of the National Health and Family Planning Commission, 2016). ART coverage rate increased from 66.2% in December 2015 (NCAIDS et al., 2016) to 86.6% by October 30, 2019 (Wang et al., 2019). The emergence of HIV drug resistance (HIVDR) has been inevitable with the rapid increase in ART coverage and is a major threat to its successful adoption. A recent systematic review revealed that transmitted drug resistance (TDR) has risen in China since 2012, which has mostly been driven by non-nucleoside reverse transcriptase inhibitor (NNRTI) resistance (Zuo et al., 2020).

Monitoring TDR is essential for preventing ART failure and limiting the spread of TDR. The prevalence of TDR in low- and middle-income countries has been estimated—usually in a small sample (*n* ≤ 47)—using the HIVDR threshold survey of newly diagnosed HIV infections recommended by the WHO in 2003 (Bennett et al., 2008). The results of these surveys showed that the rate of TDR in many regions of China was low (<5%) (Guo et al., 2015; Sun et al., 2017) or moderate (5–15%) before 2017 (Chen et al., 2017; Lu et al., 2017). Although this survey was easy to implement, subtle and important information on TDR may have been overlooked owing to the extremely low sampling rate.

Molecular network analysis based on *pol* gene sequences from HIVDR detection can be used to reconstruct the viral transmission network, which can clarify the pattern of HIV transmission and guide the development of prevention and control measures (Wertheim et al., 2014; Zhao et al., 2021). However, molecular network technology has rarely been used to explore HIVDR transmission in China. The *pol* gene sequences of men who have sex with men (MSM) receiving ART in Sichuan province were used to construct a molecular network, which showed that HIVDR was less likely to fall into clusters (Yuan et al., 2019). Although in-depth sampling recently carried out in Beijing (9,203 sequences) and Shenzhen (10,378 sequences) revealed a TDR prevalence of 4.1 and 6.0%, respectively (Ye et al., 2020; Zhang et al., 2021), detailed information on TDR transmission and their characteristics is lacking.

Shenyang is the largest city in northeastern China and the capital of Liaoning province, which has a moderate HIV prevalence [>10,000 people living with HIV (PLWH)] (Wu et al., 2019). The number of PLWH in Shenyang increased from 3,000 in 2015 to nearly 7,000 in 2019, while the ART coverage rate increased from 60 to 85% [unpublished data from Shenyang Center for Disease Control and Prevention (CDC)]. MSM account for most newly diagnosed HIV infections (80.8%) in Shenyang (Jinping et al., 2018); and MSM in Shenyang are connected to MSM in other cities in China (Han et al., 2013; Vrancken et al., 2020), making it likely that the TDR epidemic will spread to surrounding cities including Beijing. The overall prevalence of TDR in Shenyang was low (<5%) in 2012 (Zhao et al., 2015). Since then, there have been no new epidemiologic data. We therefore retrospectively collected and analyzed data on TDR in all newly diagnosed HIV-infected cases in Shenyang from 2016 to 2019, and used molecular network analysis to establish TDR patterns since the ART policy was updated.

## Materials and Methods

### Study Subjects

This study included all individuals in Shenyang who were newly diagnosed with HIV infection from 2016 to 2019. Demographic information including sex, ethnicity, age, marital status, and education level and clinical information including HIV infection route as well as available HIV-positive cryopreserved plasma samples at diagnosis were obtained from Shenyang CDC and the First Affiliated Hospital of China Medical University. All individuals self-identified as ART-naïve before HIV diagnosis. The study was approved by the Institutional Review Board of China Medical University.

### Subtyping and HIV Drug Resistance Analysis

Viral RNA was extracted from cryopreserved plasma samples from patients. The entire protease-encoding sequence and part of the reverse transcriptase sequence (HXB2: 2253–3318) in the HIV *pol* gene were amplified using a previously described method (Zhao et al., 2011). A neighbor-joining (NJ) tree including major subtypes (A-to-K) plus some common recombinants reference sequences (such as CRF01_AE, CRF07_BC, CRF08_BC, CRF55_01B, CRF59_01B, and CRF65_cpx) downloaded from the Los Alamos database^1^ was constructed using MEGA v7.0.14 (Kumar et al., 2016) under the Kimura two-parameter model with 1000 bootstrap replicates. HIV-1 subtype was determined based on the NJ tree with a bootstrap value >70 (Han et al., 2013) and were validated using HIV Subtyping Program of the Stanford University HIV Drug Resistance Database (Liu and Shafer, 2006). Recombination Identification Program (RIP) v3.0 (Siepel et al., 1995) was used to identify potential recombinants of the sequences that could not be identified as a subtype based on the NJ tree. TDR was determined using the genotypic resistance interpretation algorithm of the Stanford University HIV Drug Resistance Database (Liu and Shafer, 2006). Mutations that could lead to low or higher resistance were judged to be TDR.

### Inferring the Molecular Network

Molecular networks were inferred based on pairwise nucleotide genetic distance (Tamura-Nei 93 substitution model) using HIV-TRACE (Kosakovsky Pond et al., 2018). Similar to the previous analysis, the optimal genetic distance threshold was identified to construct the molecular network through sensitivity analysis because the maximum number of molecular clusters could be generated at this threshold (Zhao et al., 2021). Only clusters containing ≥3 individuals with the same TDR mutation were selected for the HIVDR transmission cluster. The networks were visualized with Cytoscape v3.7.2 (Shannon et al., 2003).

### Statistical Analyses

The chi-squared test was used to compare demographic characteristics between groups. *P*-values < 0.05 were considered statistically significant and those <0.1 were considered marginally significant. All statistical analyses were performed using SPSS v25.0 software (SPSS Inc., Chicago, IL, United States). Frequency distributions of TDR, mutations, and drug sensitivity were visualized using Prism v7.04 (GraphPad, La Jolla, CA, United States).

## Results

### Study Population

A total of 2,882/3,272 sequences (88.1%) were obtained after excluding 162 individuals (4.7%) without available plasma samples. The overall distribution of subtypes was 70.0% CRF01_AE (2019/2882), 18.3% CRF07_BC (526/2882), 4.6% subtype B (132/2882), and 3.0% (88/2882) other subtypes including CRF55_01B (*n* = 21), CRF59_01B (*n* = 18), CRF08_BC (*n* = 15), CRF65_cpx (*n* = 7), C (*n* = 7), CRF67_01B (*n* = 6), CRF68_01B (*n* = 4), CRF02_AG (*n* = 3), F1 (*n* = 2), CRF85_BC (*n* = 1), CRF06_cpx (*n* = 1), CRF33_01B (*n* = 1), CRF103_01B (*n* = 1), and subtype A (*n* = 1). Additionally, unique recombinant forms were detected in 4.1% of samples (117/2882).

Among the 2,882 cases, 93.2% (2686/2882) were male and 86.1% (2482/2882) were of Han ethnicity. The median age was 32 years [interquartile range (IQR) = 26–45 years]. Most subjects were unmarried (62.7%, 1808/2882), and 70.1% (2019/2882) had senior high school education or higher; 81.7% (2356/2882) were MSM. Individuals infected with CRF07_BC were younger (<30 years: 48.5%, *p* < 0.0001), unmarried (72.1%, *p* < 0.0001) MSM (86.7%, *p* < 0.0001), and had a senior high school education (76.6%, *p* = 0.0004) (Table 1).

**TABLE 1:** The demographic and clinical characteristics of the people living with HIV in four groups.

Characteristics	All (*n* = 2882)	%	CRF01_AE (*n* = 2019, %)	CRF07_BC (*n* = 526, %)	B (*n* = 132, %)	Others (*n* = 205, %)	χ ^2^	*p*-value
**Gender**								
Male	2686	93.2	1891 (93.7)	498 (94.7)	117 (88.6)	180 (87.8)	16.236	0.001
Female	196	6.8	128 (6.3)	28 (5.3)	15 (11.4)	25 (12.2)		
**Race/ethnicity**								
Han	2482	86.1	1741 (86.2)	448 (85.2)	120 (90.9)	173 (84.4)	3.915	0.688
Others	399	13.8	277 (13.7)	78 (14.8)	12 (9.1)	32 (15.6)		
NA	1	0.0	1 (0.0)	0 (0.0)	0 (0.0)	2 (1.0)		
**Age at enrollment (years)**								
<30	1128	39.1	735 (36.4)	255 (48.5)	57 (43.2)	81 (39.5)	28.205	<0.0001
≥30	1752	60.8	1283 (63.5)	270 (51.3)	75 (56.8)	124 (60.5)		
NA	2	0.1	1 (0.0)	1 (0.2)	0 (0.0)	0 (0.0)		
**Marital status**								
Unmarried	1808	62.7	1226 (60.7)	379 (72.1)	82 (62.1)	121 (59.0)	29.140	<0.0001
Married/Divorced/Widower	1067	37.0	789 (39.1)	146 (27.8)	50 (37.9)	82 (40.0)		
NA	7	0.2	4 (0.2)	1 (0.2)	0 (0.0)	2 (1.0)		
**Education level**								
Junior high school and below	853	29.6	619 (30.7)	122 (23.2)	47 (35.6)	65 (31.7)	18.231	0.0004
Senior high school and above	2019	70.1	1392 (68.9)	403 (76.6)	85 (64.4)	139 (67.8)		
NA	10	0.3	8 (0.4)	1 (0.2)	0 (0.0)	1 (0.5)		
**Infection route**								
MSM	2356	81.7	1650 (81.7)	456 (86.7)	98 (74.2)	152 (74.1)	45.209	<0.0001
HST	434	15.1	304 (15.1)	54 (10.3)	27 (20.5)	49 (23.9)		
IDU	43	1.5	38 (1.9)	4 (0.8)	0 (0.0)	1 (0.5)		
Others/NA	49	1.7	27 (1.3)	12 (2.3)	7 (5.3)	3 (1.5)		

*ART, antiretroviral therapy; MSM, men who have sex with men; HST, heterosexual transmission; IDU, injected drug user; NA, not available.*

### Transmitted Drug Resistance Prevalence and Patterns

**FIGURE 1:**
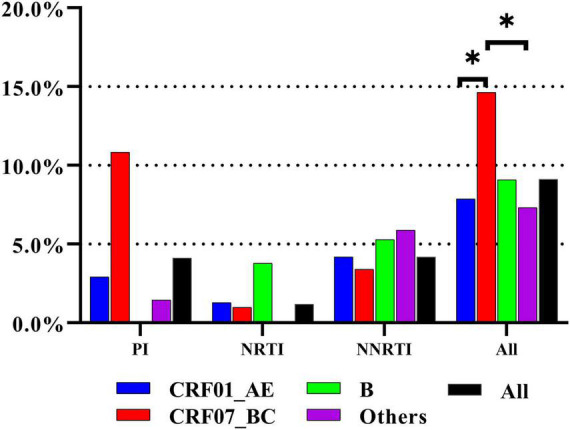
The prevalence of TDR to three class of antiretroviral drugs in different subtypes. The prevalence of TDR was highest in CRF07_BC. * represents statistically significant.

The overall prevalence of TDR was 9.1% [95% confidence interval (CI): 8.1–10.2%]. The prevalence of protease inhibitor (PI), NRTI, and NNRTI mutations was 4.1% (95% CI: 3.4–4.9%), 1.2% (95% CI: 0.9–1.7%), and 4.2% (95% CI: 3.5–5.0%), respectively (Figure 1). Among all HIV subtypes, the prevalence of TDR was highest in CRF07_BC [14.6% (95% CI: 11.7–18.0%)], which was significantly or marginally significantly higher than in other subtypes [CRF01_AE: 7.9% (95% CI: 6.7–9.1%), *p* < 0.0001; subtype B: 9.1% (95% CI: 4.8–15.3%), *p* = 0.0990; other: 7.3% (95% CI: 4.2–11.8%), *p* = 0.0066] (Figure 1).

**FIGURE 2:**
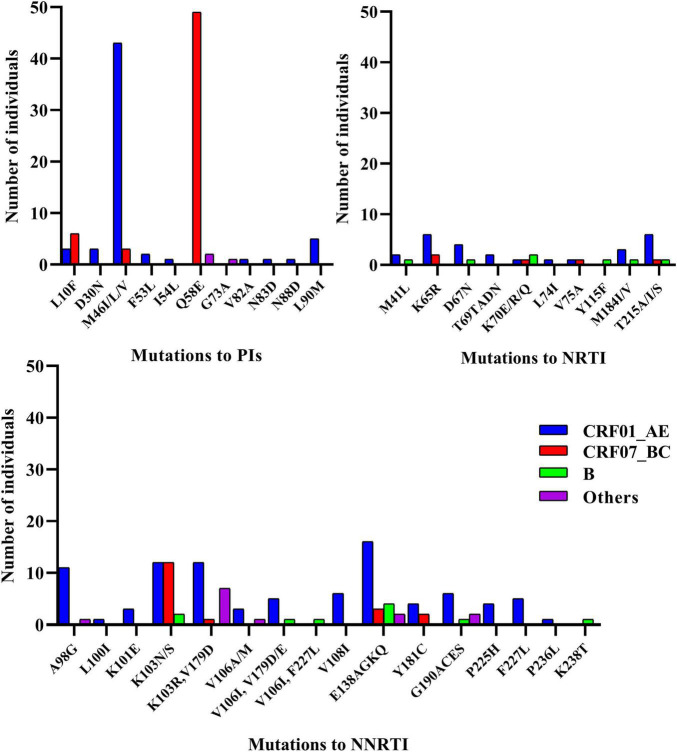
The number of TDR mutations according to gene position.

Transmitted drug resistance mutations detected in more than 10 cases included PI [Q58E (*n* = 51) and M46ILV (*n* = 46)] and NNRTI [K103N (*n* = 26), E138AGKQ (*n* = 25), K103R/V179D (*n* = 20), and A98G (*n* = 12)] mutations. Eleven TDR strains harbored dual-class mutations (NRTI + NNRTI, *n* = 7; PI + NRTI, *n* = 3; and PI + NNRTI, *n* = 1), and all strains of CRF65_cpx (*n* = 7) carried a combination of the K103R and V179D mutations (Figure 2).

**FIGURE 3:**
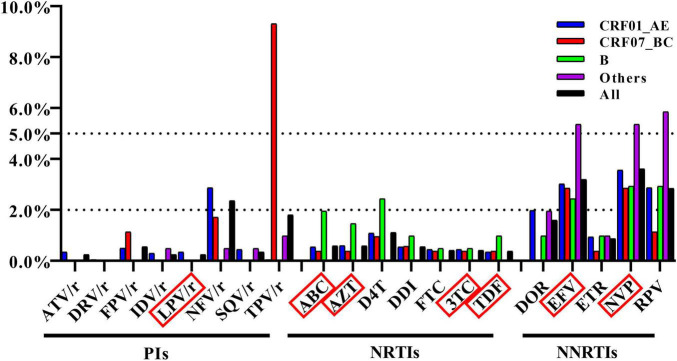
Frequency of TDR across antiretroviral drugs. The antiretroviral drugs in red box were in the list of recommended antiretroviral drugs in China.

We examined the influence of TDR on the sensitivity to ART drugs and found that NNRTIs were most affected. The top three affected drugs were nevirapine (NVP) [3.6%, (95% CI: 3.0–4.4%)], efavirenz (EFV) [3.2%, (95% CI: 2.6–3.9%)], and rilpivirine (RPV) [2.8%, (95% CI: 2.3–3.5%)]. Additionally, we found that TDR to tipranavir/ritonavir (TPV/r) in CRF07_BC strain was extremely high [9.3%, (95% CI: 7.0–12.1%)], which was mainly related to the low-level resistance of TPV/r conferred by the high frequency of Q58E (Figure 3).

### Transmission of Transmitted Drug Resistance

**FIGURE 4:**
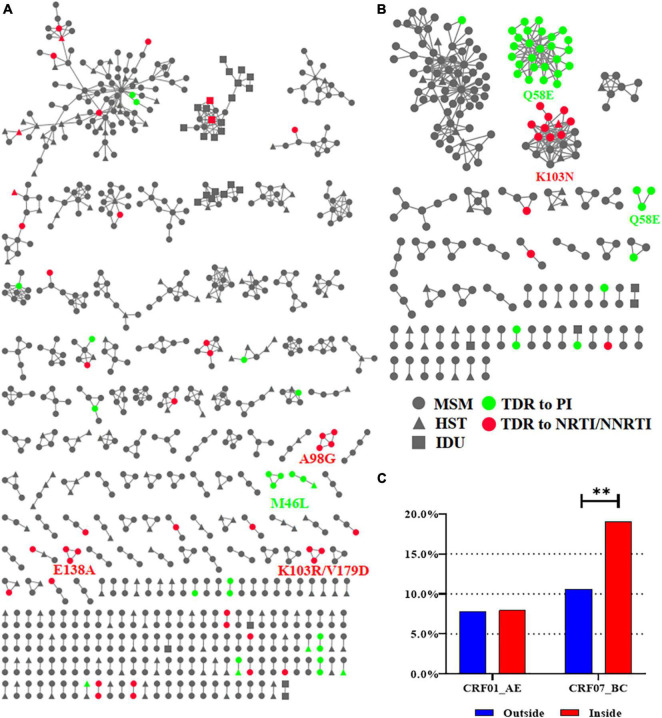
The CRF01_AE and CRF07_BC molecular networks. **(A)** The molecular network of CRF01_AE. **(B)** The molecular network of CRF07_BC. **(C)** Comparison of the proportion of TDR inside and outside the network. Only clusters containing ≥3 individuals with the same TDR mutation were identified for the HIVDR transmission cluster and were labeled with the name of mutations. Two asterisks represented statistically significant. MSM, men who have sex with men; HST, heterosexual transmission; IDU, injected drug user; TDR, transmitted drug resistance; PI, protease inhibitor; NRTI, nucleoside reverse transcriptase inhibitor; NNRTI, non-nucleoside reverse transcriptase inhibitor.

To explore TDR spread, we constructed CRF01_AE and CRF07_BC molecular networks as they harbored nearly all (*n* > 3) of the detected high-frequency TDR mutations (89.7%, 236/263) (Zhao et al., 2021). We inferred 232 CRF01_AE clusters (size range: 2–99) and 55 CRF07_BC clusters (size range: 2–70) (Figure 4). The proportion of TDR within the CRF07_BC network was significantly higher than that outside the network (19.0% vs. 10.6%, *p* = 0.0067), whereas no significant difference was observed in the CRF01_AE network.

In the CRF07_BC network, there were three clusters with TDR mutations. All individuals in two of the CRF07_BC clusters carried Q58E (PI mutations, *N* = 29) and 52.6% (10/19) in the other CRF07_BC clusters carried K103N (NNRTI mutations). In the CRF01_AE network, there were five clusters with TDR mutations including two with M46L (PI mutations, *n* = 6) and one each with A98G (NNRTI mutations, *n* = 4), E138A (NNRTI mutations, *n* = 3), and K103R/V179D (NNRTI mutations, *n* = 3). Of all clusters, 96.4% (53/55) of individuals with TDR mutation in these clusters were MSM and only two (in clusters with M46L and K103N) were infected through heterosexual transmission (HST) (Figure 4).

## Discussion

In this study, we analyzed the prevalence and spread of TDR in Shenyang from 2016 to 2019. We not only identified TDR mutations but also defined TDR clusters through molecular network construction, thus providing precise targets for targeted interventions.

In this study, the overall prevalence of TDR in Shenyang was 9.1%, which is a moderate level (5–15%) and higher than the latest results reported in Beijing, Guangxi and Guangdong (Ye et al., 2020; Lan et al., 2021; Pang et al., 2021). The prevalence of TDR to NNRTI was 4.2%, which may be related to the low genetic barrier of NNRTIs and their widespread implementation as first-line regimens in China since 2016 (General Office of the National Health and Family Planning Commission, 2016). Although the prevalence of TDR to PIs was as high as 4.1%, the top two mutations (Q58E and M46ILV) to PIs mainly target nelfinavir and TPV/r and have little impact on lopinavir/ritonavir (LPV/r), which is recommended as a second-line therapy in China (Aids and Hepatitis C Professional Group, Society of Infectious Diseases, Chinese Medical Association; Chinese Center for Disease Control and Prevention, 2018). Only I54L (*n* = 1), V82A (*n* = 1), and L90M (*n* = 5) conferred low or intermediate resistance to LPV/r, suggesting that the low prevalence of these TDRs is related to the fact that LPV/r is not widely used as a second-line drug in China. Therefore, the TDR to PIs observed in this study could be overestimated.

The most important result of this study was the establishment of TDR clusters, especially in CRF07_BC. First, we found that 62.3% of CRF07_BC strains with TDR were within the network, suggesting that many CRF07_BC strains with TDR can be transmitted. Our results showed that CRF01_AE and CRF07_BC were the top two subtypes in Shenyang, and there were significant differences in demographic characteristics between people infected with these two subtypes, although MSM counted for the majority of them (>80%). The demographic data showed that most of PLWH infected with CRF07_BC were more likely to be young MSM; therefore, engagement in condomless anal sex is more likely to lead to the spread of TDR. However, the patients infected with CRF01_AE were more likely to be older (≥30 years old) and the transmission routes were more complex. The three clusters with TDR carried Q58E and K103N, which were the top two mutations in CRF07_BC. Q58E does not affect the sensitivity of the strain to LPV/r (only PIs are used in the second-line setting in China), therefore it can serve as a marker for transmission of a particular CRF07_BC quasispecies. K103N is known to confer high-level resistance to EFV and NVP, both of which are included in the list of free ART drugs in China (Zuo et al., 2020). Because of the low genetic barrier of NNRTI drugs, K103N/S is the predominant mutation in all countries reporting HIVDR data according to the WHO (World Health Organization, 2019) and a study conducted in China (Zuo et al., 2020). Therefore, it was not surprising that K103N was the top NNRTI mutation and was detected in CRF01_AE (*n* = 12), CRF07_BC (*n* = 12), and subtype B (*n* = 2) in this study. However, K103N was present only in the CRF07_BC cluster, suggesting that this strain with the K103N mutation was transmitted and can be further propagated. This is the first report of the recent concentrated spread of TDR among newly diagnosed cases of HIV infection in China. We also collected demographic and ART information on individuals in the cluster with K103N; nine out of 10 cases were MSM and one case was a male infected through HST. The median age was 25 years (IQR = 23–27 years). Eight of these individuals received ART and achieved virus suppression. However, of the two cases diagnosed in 2019, one did not receive ART and one was lost to follow-up. The growth of this cluster should be monitored as there is a risk for the emergence of TDR.

Second, we identified five small clusters (*n* < 5) with TDR in CRF01_AE. 26 M46I and 14 M46L mutations were detected in CRF01_AE. However, only two small clusters with M46L (*n* = 6) were obtained, suggesting that M46I/L is a natural polymorphic mutation of CRF01_AE. The remaining three clusters carried NNRTI-related TDR mutations (A98G, K103R/V179D, and E138A), which may confer different levels of resistance to NVP, EFV, and RPV. Our results suggest that NNRTI-related TDR mutations have been transmitted in CRF01_AE and may be at the early stages of transmission because of the small cluster size (*n* < 5). All 10 cases in these three clusters were MSM, and the median age was 25 years (IQR = 25–30 years); nine individuals received ART and developed virus suppression and one individual with A98G was lost to follow-up. Notably, the same number of strains with K103N was detected in CRF01_AE (*n* = 12) and CRF07_BC (*n* = 12) but the CRF01_AE cluster with K103N was not in the network, implying that these strains had not been transmitted. Although the latest studies have applied molecular network technology to explore the transmission of TDR, they did not identify molecular clusters with same TDR due to the lack of sampling depth or enough time span (Lan et al., 2021; Pang et al., 2021; Zhang et al., 2021). Our study constructed an HIV molecular network of the whole population in Shenyang through in-depth sampling, obtained the actual molecular clusters with TDR, and provided accurate targets for targeted intervention.

The discovery of TDR clusters in this study indicates that real-time molecular network monitoring based on *pol* sequences from all newly diagnosed cases of HIV infection is essential for precisely targeted interventions to halt the spread of TDR. A study conducted in Canada in 2016 reported the implementation of an automated near–real-time monitoring system to monitor the outbreak of an HIV TDR cluster (eight cases) and assessed the effectiveness of public health action (Poon et al., 2016). However, it is not easy to establish a real-time monitoring system in low- and middle-income countries. In China, TDR testing is not a routine practice and HIVDR testing is recommended only when the viral load after ART is not ideal or when ART fails (Aids and Hepatitis C Professional Group, Society of Infectious Diseases, Chinese Medical Association; Chinese Center for Disease Control and Prevention, 2018) because of the high cost and technical requirements. As an alternative strategy, based on available drug resistance data, close follow-up of PLWH with TDR and HIV screening and TDR testing of the high-risk individuals who have close contacts with them should be conducted. However, in provinces or cities with a better economy such as Beijing and Shenzhen, baseline TDR testing is recommended.

Some other findings of this study are worth mentioning. First, V179D and K103R/V179D occur naturally in HIV-1 CRF65_cpx (Liu et al., 2020); this was confirmed by our finding that all 7 individuals infected with CRF65_cpx in this study also carried K103R/V179D. Second, some TDR mutations do not affect the ART sensitivity of the strain when they are present in isolation but can confer low or intermediate resistance or increase the existing TDR level in combination with other mutations. Such mutations that were detected in this study mostly targeted NNRTIs, including K103R/V179D, V106I/V179DE, V106I/F227L, and E138AG/V179DE. At last, although most molecular clusters were dominated by MSM, most molecular clusters, especially some molecular clusters with TDR, contained some PLWH infected through HST. It suggested that they may be a potential bridge for transmitting TDR to other high-risk populations and should be paid attention to.

There were some limitations to this study. First, the links between individuals in clusters did not represent true transmission relationships; the actual sources of TDR need to be identified through epidemiologic investigations. Second, the lack of high-risk behavioral information makes it impossible to establish sexual contact networks of individuals with TDR, thus limiting the possibility of behavioral interventions to prevent the spread of TDR.

## Conclusion

Transmitted drug resistance was moderately prevalent (9.1%) in Shenyang from 2016 to 2019, and there was evidence of spread of the CRF07_BC and CRF01_AE strains with TDR. This study provides a good example of monitoring TDR and identifying precise targets for intervention by molecular network analysis.

## Data Availability Statement

The original contributions presented in the study are included in the article/Supplementary Material, further inquiries can be directed to the corresponding author/s.

## Ethics Statement

The studies involving human participants were reviewed and approved by the Institutional Review Board of China Medical University. The ethics committee waived the requirement of written informed consent for participation.

## Author Contributions

HS and XH conceived the study. WS, XD, XL, LuW, JL, HD, and ZC collected samples and epidemiology data. BZ, LiW, and YQ conducted the experiments and collected the data. BZ and MK analyzed the results. BZ drafted the manuscript. XH reviewed and edited the manuscript. All authors read and approved the final manuscript.

## Conflict of Interest

The authors declare that the research was conducted in the absence of any commercial or financial relationships that could be construed as a potential conflict of interest.

## Publisher’s Note

All claims expressed in this article are solely those of the authors and do not necessarily represent those of their affiliated organizations, or those of the publisher, the editors and the reviewers. Any product that may be evaluated in this article, or claim that may be made by its manufacturer, is not guaranteed or endorsed by the publisher.

## Footnotes

1
http://www.hiv.lanl.gov/
